# Bilateral retinal implants for improving visual restoration: a simulated bionic vision study

**DOI:** 10.1007/s11517-026-03513-3

**Published:** 2026-02-11

**Authors:** Pablo Rodriguez-Miguez, Pablo Ramon-Soria, Alejandro Barriga-Rivera

**Affiliations:** 1 Departamento de Física Aplicada III. Escuela Técnica Superior de Ingeniería, Universidad de Sevilla, Camino de los Descubrimientos, s/n, Sevilla, 41092 Spain; 2 School of Biomedical Engineering, University of Sydney, Sydney, Australia

**Keywords:** Visual prosthetics, Bionic, Eye, Binocular retinal implant, Virtual reality, Neural stimulation

## Abstract

Degenerative diseases of the retina produce a severe deterioration in visual perception. Visual prostheses enable partial vision restoration by eliciting phosphenes (perceived spots of light) through electrical stimulation. By using augmented reality (AR) to simulate bionic vision (SBV), this study aims to provide insights on the potential benefits of bilateral retinal implants. While monocular implants have been extensively studied, research on binocular implants remains limited. Twenty-four volunteers participated in this study, and were divided into two groups, SBV-monocular and SBV-binocular groups; both experienced phosphene-based visual simulations of the scene using 120 phosphenes per implant. A control group perceived the scene directly through the cameras of the AR headset. Participants completed five tasks to assess their orientation, mobility, and pattern recognition capacities. SBV-binocular subjects outperformed in spatial perception and mobility tasks (*p*-value < 0.05), but not in pattern recognition tasks. All SBV groups perform significantly worse than the control group. The findings suggest that the bilateral implants’ enhanced field of view and stereoscopic depth cues afforded by bilateral implants were shown to support hand-eye coordination and spatial navigation tasks. However, relevant benefits were not demonstrated over monocular implants in detecting fine details in static tasks. This may be attributed to the absence of vergence control, the capacity to shift phosphene position in response to natural eye movements, in the bilateral configuration.

**Graphical abstract:**

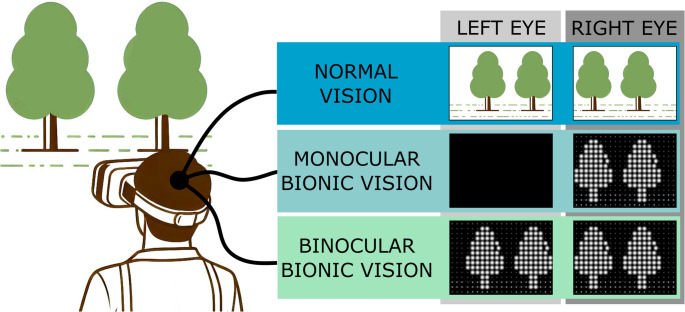

## Introduction

Vision loss caused by retinal degenerative diseases such as retinitis pigmentosa can be treated by electrostimulation of the surviving retinal neurons, i.e., using retinal neuroprostheses [1–3]. These electronic devices, broadly known as ‘bionic eyes’, allow the restoration of eyesight with a low-resolution vision characterized by perceptual clusters of visual sensations elicited from the implanted electrodes, typically referred to as phosphenes [4]. Although photoreceptors are typically defective or damaged in these conditions, up to 90% of retinal neurons, including the ganglion cell layer, are still functional in many cases, and can be used to deliver visual information to the brain. Retinal prostheses are typically placed in either, the epirretinal, the subretinal, or the suprachoroidal space. In contrast, other bionic devices target more central structures of the visual pathway such as the optic nerve lateral geniculate nucleus, or the visual cortex. Among retinal technologies, few have received regulatory approval for clinical use: the Argus II (Second Sight, Sylmar, USA), an epiretinal implant, and the Alpha AMS (Retinal Implant AG, Reutlingen, Germany) a subretinal implant whose development was later acquired by Pixium Vision. Current implant models offer limited visual performance with visual acuity typically bellow the legally blind limit of 20/200, fields of view ranging from 20 to 30 degrees, and relatively low electrode count (between 60 and 378) [2, 5, 6]. Retinal prostheses can artificially activate the retinal ganglion cells by delivering patterned electric current from a retinal electrode array, thus conveying neural messages that propagate downstream across the visual system to generate visual sensations [7]. However, three main factors hamper the performance of retinal prosthetics: (1) a relatively low electrode count limits the resolution of the implants, and consequently the regained visual acuity, (2) the extent and location of the implanted electrode array limits the field of view, and (3) the inability to convey meaningful neural messages from a retinal implant compromises the stability of the visual percepts [8, 9]. Although the vision thus regained is somehow rudimentary, it may assist users with basic tasks like navigation of mobility [10]. However, it remains insufficient for visually demanding activities such as recognition of faces, manipulation of fine objects, or reading among others [11, 12]. In addition to the low resolution due to the limitation in the number of electrodes implanted and the narrow field of view provided by a relatively small retinal implant [13], bionic vision quality is also limited by patient-related factors that prevent these tasks from being executed correctly. Age at implantation is one of the key intrinsic factors: younger subjects have a greater capacity for healing after surgery and exhibit better adaptation to the implant due to an increase neuroplastic capacity (the ability of neurons to grow and reorganize) [14, 15]. Another factor refers to the stage of retinal degeneration. Thus, a greater number of intact neuronal circuits may improve the encoding and interpretation of artificial neural signals [15].

As mentioned previously, one of the most significant limitations that compromises the performance of bionic vision is the limited field of view achieved with a retinal prosthesis. There have been many research efforts aiming to expand the number of phosphenes [16–19]. These strategies would increase the resolution, but not the field of view unless they cover larger retinal areas. This increase in resolution is particularly effective in object recognition tasks, where the target remains approximately steady and show significant contrast levels relative to the background [19]. It has to be noted that movement itself is a key factor in finding and distinguishing an object within a given visual scene, as it can compensate for the lack of resolution [20]. However, in those tasks where the perception of moving objects is required, a good resolution along with an extended field of view provides the optimal configuration in artificial prosthetic vision [13].

An important visual feature that is lost after the implementation of a bionic eye is the perception of depth or 3-dimensional (3D) vision. Owing to the positioning of the human eyes within the same planar field, the retinas project identical images from two marginally different angles, a phenomenon known as binocular disparity. The eyes can move in a coordinated fashion to allow the image of an object to form over corresponding points of the two retinas, a process called vergence. The disparities among the two perceived images are interpreted in the brain resulting in the perception of depth or distance [21]. This ability, called stereoscopic vision, has been analyzed previously [22, 23] and tried to be simulate with artificial retinas to be used in robotic applications [21, 24, 25], but not in the field of retinal prosthetics. In physiological vision, disparities as small as approximately 10 arcseconds can be sufficient for depth perception, well below the 1-degree typical field of view covered by a single phosphene in a retinal implant.

Adding a second bionic eye could have the potential to be benefit the users of retinal prosthetic devices by extending both, the overall perceptual resolution and the field of view. Note that this strategy allows to double the electrode count and to substantially increase the field of view, and therefore, has the potential to improve real-world challenges such as navigation, object recognition, and 3D perception among others. However, to the knowledge of the authors, this possibility has not been yet explored unlike in the cochlear implant, where bilateral implants have demonstrated extraordinary benefits [26, 27]. Cochlear implants, or the ‘bionic ears’, have been used to restore audition for several decades with excellent results worldwide [28]. These are also neuroprostheses that, by electrically stimulating the auditory nerves with electric pulses *via* electrode array implanted into the inner ear, allow for the restoration of hearing [29]. Numerous studies have been carried out to assess the performance of a single cochlear implant [30–33], and bilateral implants [34–37]. It has been shown that the addition of second implant improves, not only the speech intelligibility but also the ability to locate sound sources, as interaural differences (or the so-called ‘head shadow’) are perceived [38, 39]. Consequently, given the excellent results obtained from bilateral bionic ears, it can be expected that bilateral retinal implants can also help improving the performance of bionic eyes. Several factors may explain why this approach remains poorly explored in bionic vision: (1) the cost of a double implant, (2) the technical challenges related to the operation of two implants simultaneously (synchronization, interocular alignment, etc.), and (3) the ethical considerations that may arise from early-stage trials among others.

Clinical studies on retinal prosthesis have been conducted to explore the performance of monocular implants [10, 40]. Typically, clinical trials are approved once sufficient scientific evidence is available. Thus, many aspects of the implants have been previously explored in the laboratory using animal or computational models [41–49]. In order to assess the performance of some of these electrical stimulation strategies, the effectiveness of different visual rehabilitation approaches, or to determine their potential limitations when using real implants in humans prior to clinical trials, researches have employed simulated prosthetic vision, as it provides relevant psychophysical information [50, 51]. Typically, these studies employ augmented or virtual reality (AR and VR respectively) systems to mimic the vision as regained by an implant. In previous experiments, a matrix of virtual phosphenes has been mapped to transform the video stream from camera placed near the eye while groups of volunteers were asked to perform tasks of different types: letter or pattern recognition, or immersive orientation and mobility task among others [52, 53]. More recently, this simulated prosthetic vision has been integrated within a video game environment [52, 54, 55]. These studies, like previous clinical trials, have only considered monocular implants, which leaves a significant gap in our understanding. This study investigates whether bilateral implants can improve visual restoration through visual prosthetics. Current virtual and AR technologies allow for the recreation of 3D vision in many applications [56–60]. Here we present a psychophysical study wherein volunteers were asked to perform different tasks under monocular and binocular simulated prosthetic vision using a customized augmented reality (AR) system. The goal of this study is therefore to determine whether bilateral retinal prosthesis, as in the case of cochlear implants [61], improves performance metrics, particularly regarding the acquisition of stereoscopic vision. This study offers a safe, affordable and scalable method to explore the promise of bilateral retinal implants while avoiding the surgical, economic and technical limitations that held back their development. If proven effective, bilateral implants may bring new hope to those seeking to regain vision.

## Methods

An AR-based simulation of bionic vision was used to assess the performance differences between monocular and binocular retinal implants. For that purpose, a head-mounted device (HMD) was used to provide a simulated bionic vision (SBV), both monocular and binocular.

This study was approved by the Research Ethics Committee of the Universidad of Sevilla. Prior to the commencement of the experiments, volunteers were tested for normal vision as described below. Illumination was controlled by employing indoor artificial illumination only in a closed room to ensure similar conditions throughout the experiments.

A total of five tasks were investigated over two days to account for participant familiarization. The first two tasks were completed during the first day of participation, and the remaining three tasks during the second one. Each of the tests are described in the section ‘Experimental Procedure’.

### Participants

Normally sighted subjects aged over 18 years were invited to participate in the study. A standard Snellen visual acuity test was performed prior to admitting a volunteer to ensure 20/20 vision. The chart was printed on an A4 size white paper. Volunteers remained seated 6 m away from the chart during the test, and subjects with conditions (visual acuity bellow 20/20, cataracts, etc.) that deterred them from performing the experimental tasks or who had previously participated in a simulated bionic vision experiment were excluded. No participant had previous relationship of any kind with the experimenters. The 24 volunteers (21 men and 3 women), aged between 32 and 60 registered in this study read and signed an informed consent form prior to enrolling. Note the sample was gender imbalanced, and therefore sex-related conclusions cannot be drawn from this study. However, differences among men and women are not expected in this experiment. Volunteers were then randomly sorted into three groups: (1) SBV-monocular, (2) SBV-binocular, a (3) control. Ten participants were randomly assigned to each of the monocular and binocular groups, while four participants formed a control group. These experimental groups allowed to compare the performance of single-eye retinal implants (SBV-monocular group) against bilateral implants (SBV-binocular group), while having information on the optimal performance (controls). Before conducting the experiments, volunteers were given instructions on how to perform the tests using examples not included in the experiments, and were allowed to explore the experimental room with and without the AR system to get familiar with the distances. Participants were then asked to perform different tasks to assess visual acuity and 3-dimensional (3D) perception capabilities. The control group performed the tasks under non-phosphenized vision provided by the headset. In doing so, the passthrough mode was used to display the visual scene as captured by the cameras of the AR system. Note that the field of view remained constant in all groups. Pixel-wise, the resolution of the image processed before and after the phosphenized conversion remained the same. The purpose of the control group was to provide for a reference measurement of the performance of phosphenized vision. The fact of using the headset instead of using direct vision attempts to set the same comfort conditions among the groups, reduce the FoV and account for latencies in the rendering of the scenes (either direct passthrough of the cameras or phosphenized image).

### Equipment

An AR system was designed to simulate bionic vision, both as obtained from a single retinal implant, and from bilateral retinal implants. This system employs a Valve Index^®^ VR Kit (Valve Corporation, Washington, USA) headset to transform the images obtained from an array of cameras into a phosphenized representation. For that purpose, the VR kit was connected to a computer (X299X AORUS^®^ MASTER running Windows^®^ 10 64 bits with an Intel^®^ i9 processor and NVIDIA^®^ GeForce RTX 2080 Ti GPU) executing custom simulation software developed in C + + for the Unreal Engine>^®^ platform (Epic Games Inc., North Carolina, USA), which allows real time rendering of the target simulations. This software was developed by the researchers inspired by previous reports [4, 7].

**Fig. 1:**
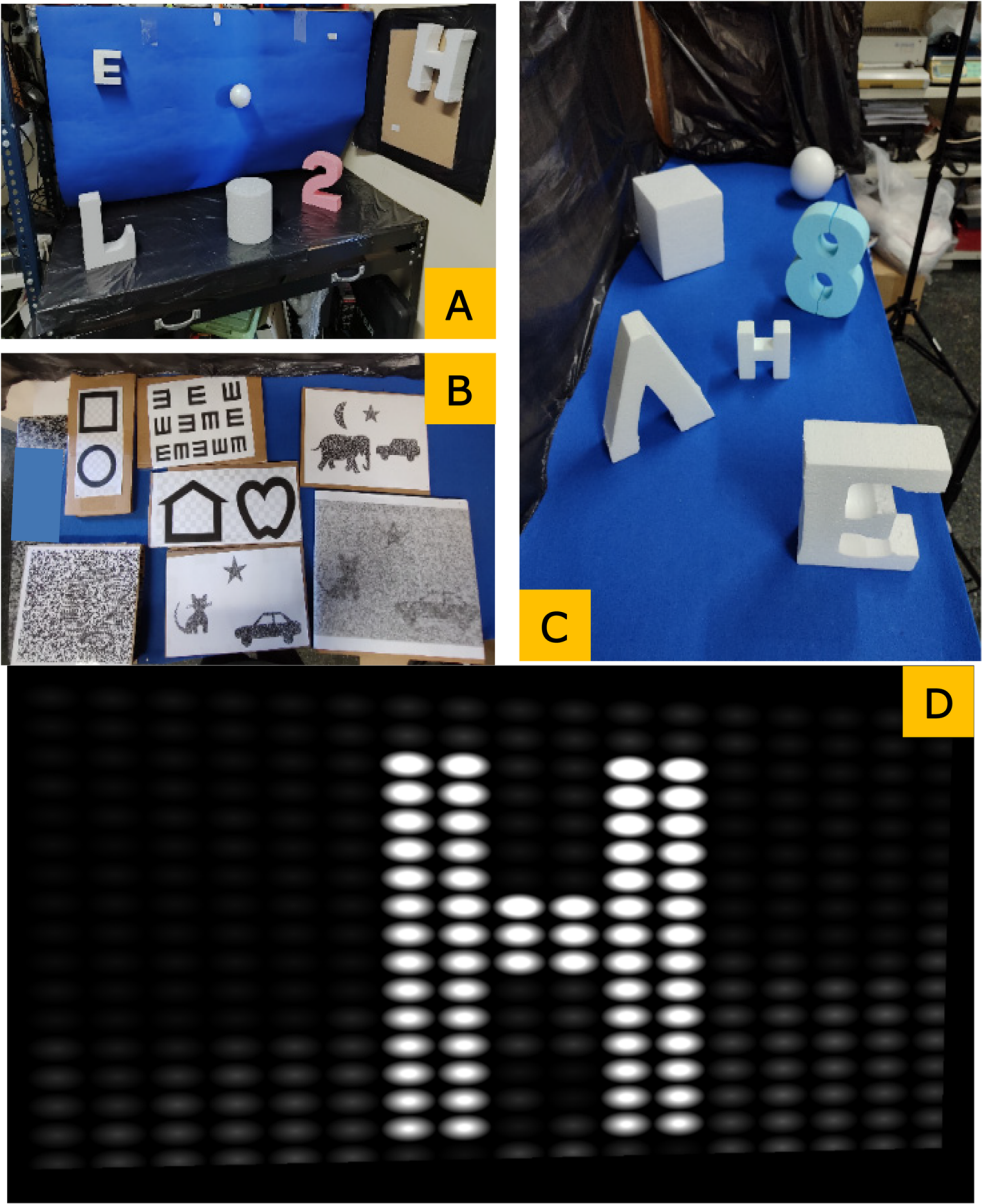
Example of experimental set-ups used during the experimentation for (**A**) hand-eye coordination for spatial shape recognition (TASK 1), (**B**) for 2-dimentional shape recognition (TASK 4), and (**C**) and for static stereoscopic vision (TASK 5). These images were taken from the position where the volunteers performed the tests. Example of a phosphenized representation of the 3D object letter ‘H’(**D**)

To study hand-eye coordination, 3D objects such as letters, numbers, spheres or cubes made from polystyrene foam, and lightweight plastic balls were used, as shown in Fig. 1A and B. A screen (Samsung^®^ model U28E590D 32-inch) was employed for displaying images and videos with movement, required to study other dimensions of visual perception.

Prior to starting the experiment, volunteers tailored and adjusted the VR glasses to their heads to ensure it remained stable. Next, they were asked to adjust the lenses of the system to accommodate their interpupillary distance. This adjustment was carried out with the VR system in passthrough mode (visual scene presented as captured by the camera) while observing the Snellen chart to ensure normal acuity was maintained. During the experiment, volunteers performed different tasks while sitting or standing in a steady position, or while moving around the experimental room. Note a safety perimeter was established using a ribbon.

### SBV software

The software developed for this study emulated the vision obtained from monocular and binocular retinal prostheses. A total of 120 elliptical Gaussian phosphenes, with the same spatial separation, were used per implant, covering a visual field of 50 ± 2 degrees, as in the example shown in Fig. 1D. This number lies between the number of electrodes included in the Argus II (60 electrodes) and the PRIMA (378 electrodes) retinal prostheses thus providing a fair representation of the current technology. Each phosphene represented a visual field of approximately 4 degrees. The shape of the phosphenes used has been extensively reported in the scientific literature for simulated bionic vision [62–64], as it is the most prevalent percept. However, phosephenes have been reported to exhibit, not only Gaussian profiles but also more complex forms such as lines or halos in suprachoroidal [65] and epiretinal implants [66].

The Valve Index^®^ HMD is provisioned with two front facing color cameras that can be used to show the environment to the user while wearing the device. The software designed in this study modified the video stream of these cameras and processes them to simulate phosphenized scenes. To allow for real-time image processing and to guarantee a latency within real time requirements for 120 frames per second, the image processing was accelerated at the GPU of the desktop computer using OpenGL shaders.

### Experimental procedure

The volunteers recruited in this study were asked to complete five tasks involving pattern recognition and immersive orientation and mobility task to evaluate their visual acuity, hand-eye coordination and spatial orientation using a bilateral implant. For this purpose, the results obtained in each test and each group were subsequently contrasted.

- *TASK 1: Hand-eye coordination for spatial shape recognition.*

Six 3D polystyrene foam objects such as numbers, letters and geometric objects of different colors, ranging from 8 to 24 cm in height, were placed, at fixed positions, around the test room within the field of view of the participants, as shown in Fig. 1A. Volunteers were asked to pick up one by one all the objects displayed and to provide a description trying to identify each one. They were allowed to manipulate the object seeking to integrate tactile with visual perception. The time to correct identification was used as a metric in this test.

- *TASK 2: Pendulum*.

A pendulum was made by hanging a soft ball from the ceiling with a rope, 110 cm above the floor. Volunteers were requested to catch the ball with both hands while being allowed to walk freely in the experimental room. This test was performed with two different balls (a white one, 15 cm in diameter, and a black one with a diameter of 25 cm) to allow for richer experimental conditions (contrast and acuity). Each participant underwent three attempts with each ball. The average time to catch each ball with two hands was recorded as a performance metric. In addition to hand-eye coordination, this test requires spatial orientation.

- *TASK 3: Pattern recognition and movement detection.*

This test was included to assess visual acuity. For that purpose, 40 slides with four acute visual examinations of different nature were displayed on a screen. A total of four subsets of ten slides each was used. This test was to be completed *ad libitum* and therefore, slide change was done after an answer was given without taking into account the time elapsed. The number of correct observations (regardless of the subset to which they correspond) was used as a metric in this test. The first subset consisted on moving geometric figures (squares, triangles or circles), of different sizes and contrast levels relative to the background, that appeared moving from side to side following an acoustic beep signal use to alert the volunteer. Participants were asked to determine the direction of movement of the figure. If no object was perceived, the trial was repeated after re-adjusting the headset position to ensure the screen was within the field of view of the SBV system. In the subset, participants were shown Vistech gratings (VCTS), that is alternating dark and light stipes, with three different orientations and spatial frequencies comprising between fields of view from 1.5 to 15 degrees per cycle. The goal was to determine the orientation. Thirdly, a Landolt C experiment was employed to determine differences in the participants’ ability to discriminate fine features under monocular and binocular SBV. Briefly, a circle with a gap resembling the letter ‘C’ was displayed with the gap oriented in different directions to test visual acuity. Finally, a number of circles (between one to five) of different sizes and gray levels were displayed simultaneously on the screen using black or white backgrounds. Volunteers were asked to determine the number of circles shown.

- *TASK 4: Two Dimensional (2D) shape recognition.*

Based on visual acuity tests as Randot, Lea, Lang and E test, different cards with figures like letters, animals and geometric figures with different sizes and contrast with the background printed on them were given to the volunteers. The included forms and figures are shown at Fig. 1B. Subjects were allowed to manipulate the cards. The cards were handed out face down. As in the previous task, participants were asked to flip the card one by one and identify the objects as fast as possible and to determine the number of objects present in each card. A time limit of 100 s per card was established to encourage participants to engage in speed. Performance was assessed by recording the time taken to identify objects on each card. A performance metric was then established as follows: one point was awarded per object correctly identified, and an additional point was added if they succeeded in counting the total number of objects within a card (maximum points 22).

- *TASK 5: Static stereoscopic vision (depth test)*.

Six polystyrene foam 3D objects were randomly placed on fixed positions on a large table at different distances from the observer to ensure depth perception, as shown in the example of Fig. 1C. There was a minimum spacing of 10 cm between objects. The aim of this task was to determine the number of objects on the table without changing the designated standing position. The use of hands was not allowed, only being able to lean forward from the table. The aim is to measure the participant’s ability to distinguish between objects that are close to each other and at different distances from them. The number of uncounted objects was noted as a performance test.

The tasks chosen in this experiment allowed for isolating and assessing specific capabilities required in real life. For example, hand-eye coordination is essential for the manipulation of objects (cutlery, hygiene, etc.). Similarly, the pendulum task can assist with evaluating the ability of interacting with moving objects, that required to avoid a falling ball or a passing-by bicycle, while the static stereoscopic vision task provides an additional measurement of finer stereopsis, as that required for grabbing a glass without dropping it.

### Data analysis

Data were analyzed using custom scripts written in Matlab^®^ R2023b (Mathworks, Natick, U.S.A.). Performance metrics described above were compared to determine whether bilateral implants pose substantial advantages in the tasks conducted. For that purpose, the Shapiro-Wilk test was used first to discard normality of the values. Differences among the experimental groups were tested for statistical significance at the 95% confidence level using the Mann-Whitney-Wilcoxon test, a statistical test commonly employed to compare data from two independent groups. Mean values are reported with standard deviation (± SD) as the measure of variability in the main text to provide an intuitive interpretation of the data, while figures additionally display medians and interquartile ranges to show distributional features. Effect size was reported using the non-parametric measure r calculated as described in Eq. 1, where Z represents the Z-score from Mann–Whitney U test, and N is the number of observations. Outliers, defined here as values beyond 1.5 times the interquartile range, were excluded from the analysis.

1 $$\:r=\frac{Z}{\sqrt{N}}$$

## Results

As described, participants were equipped with the AR headset that simulated bionic vision as received from monocular and binocular implants. All participants performed five different tasks. The control group performed the same task with the VR, headset reproducing the environment as captured by the cameras.

### Immersive orientation and mobility

In this study, TASK 1 and TASK 2 aimed to provide an assessment of the ability of the experimental subjects to coordinate visual perception and location of objects using their hands.

**Fig. 2:**
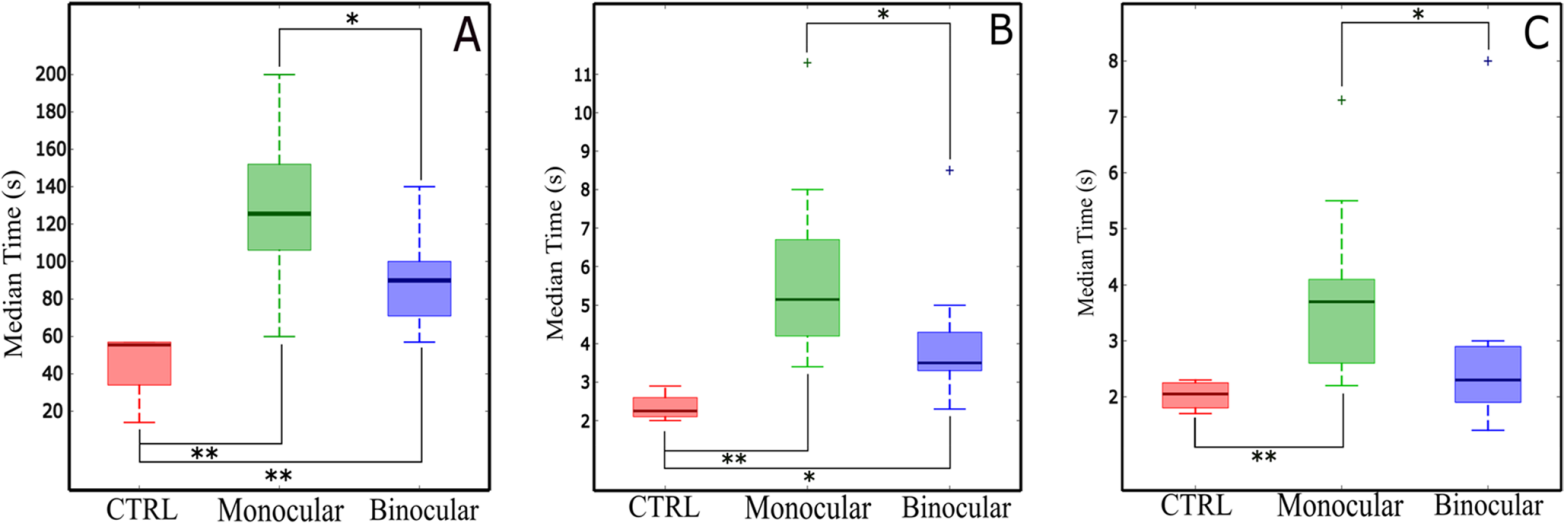
Median values of the performance metrics obtained in immersive orientation and mobility tasks. (**A**) Median time required to find and recognize presented objects during hand-eye coordination for spatial shape recognition (TASK 1). (**B**) Median value of the average time (3 attempts per subject) to catch a 25-cm-in-diameter pendulum, and a 15-cm-in-dimeter pendulum (TASK 2) (**C**). The boxes here represent the interquartile range. The whiskers comprise all recorded values but outliers. (*p-value ≤ 0.05, **p-value ≤ 0.01, Mann-Whitney-Wilcoxon, + outliers defined as 1.5 the inter-quartile range, n_CONTROL_=4, n_Monocular_=10, n_Binocular_=10)

Time required to recognize the six figures included in TASK 1 (hand-eye coordination for spatial shape recognition) showed significant statistical improvement between binocular and monocular SBV, as shown in Fig. 2A. SBV-binocular subjects performed the tasks approximately 35% faster than SBV-monocular subjects, on average, requiring 90.1 ± 22.8 and 127.7 ± 42.6 s respectively (p-value = 0.031, *r* = 0.478). The longest times required to complete the task for SBV-binocular and SBV-monocular volunteers were 140 and 200 s respectively. One participant in the control group was able to recognize all figures within 14 s, being this the fastest time recorded. Overall, the control group required 45.5 ± 21 s to complete the task. It has to be noted that during the experimentation, none of the participants enrolled in the control group required the use of their hands to complete the test. In contrast, all participants in the SBV-monocular group needed to manipulate the objects for their identification. Note also that half of the SBV-binocular volunteers did not need to use their hands for this task.

In TASK 2 volunteers were allowed to move freely around the experimental room to catch a pendulum. For the two pendula used here, SBV-binocular performed significantly faster than SBV-monocular volunteers, as illustrated in Fig. 2B and C. Again, as expected, the overall times recorded for the control group (2.35 ± 0.39 s, 2 ± 0.28 s, for large and small pendula respectively) were consistently the shortest; time differences were statistically significant with the SBV-monocular group (p-value = 0.006, *r*=−0.68, for large pendulum; p-value = 0.01, *r*=−0.74 for small pendulum). Note that for the case of the small pendulum, differences between the control and binocular groups were not statistically significant (p-value = 0.394).

Regarding the differences between both SBV groups, binocular group performed significantly faster than the monocular group, with average times of 4 ± 1.75 and 5.87 ± 2.38 s for the large pendulum (p-value = 0.028, *r* = 0.47), and 2.78 ± 1.9 and 3.88 ± 1.55 s for the small pendulum (p-value = 0.023, *r* = 0.45) respectively. It can be therefore stated that, overall, the subjects in the binocular group were able to catch the pendula 36% faster that the monocular group, but slower compared to the control group (43% slower).

Participants were indicated by the experimenter that the pendulum was in movement. After being informed that the pendulum was in motion, control group subjects moved toward it almost immediately. Half of the subjects enrolled in the SBV-binocular groups started to move seeking for the pendulum. However, 7 out of the 10 volunteers in the monocular group remained still for a relatively long period of time trying to understand the environment before initiating movement around the room. This demonstrates a deficit in their ability to orientate.

### Pattern recognition

Volunteers performed three tasks (TASK 3–5) to assess their ability in recognizing patterns of different types (figures, letters, etc.). These tasks involved recognizing 3D spatially positioned objects, 2D static figures, and motion patterns displayed on a screen.

**Fig. 3:**
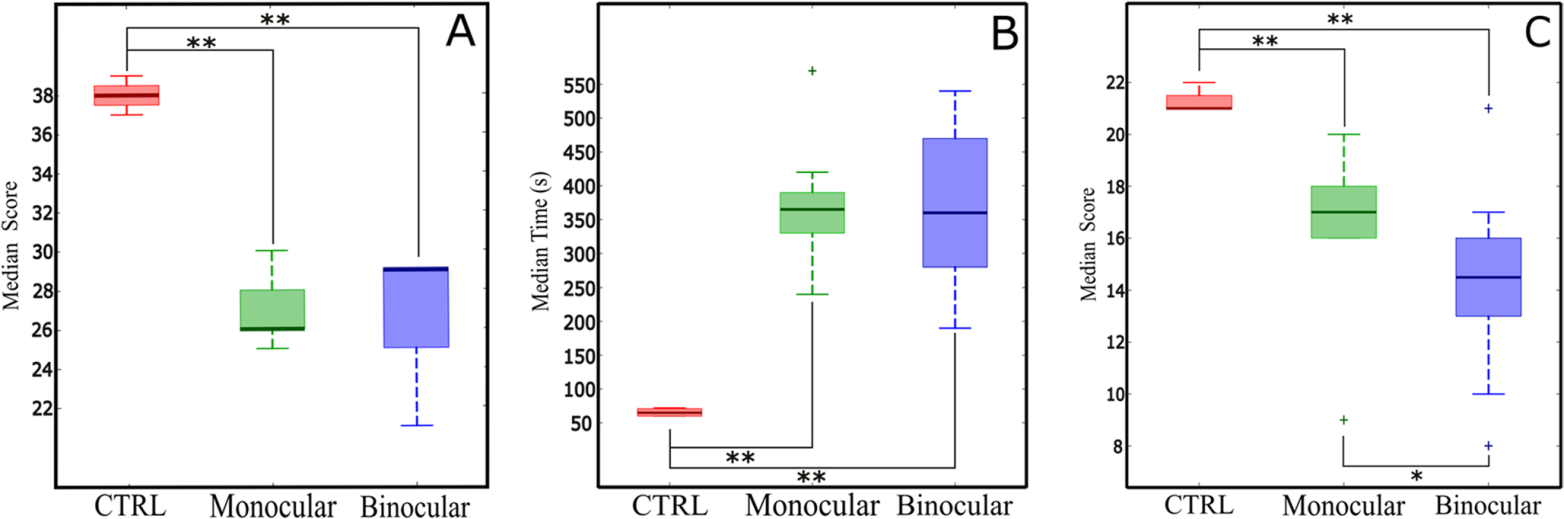
Median values of the performance metrics obtained in pattern recognition tasks. (**A**) Median of the number of correct observations made by looking at the screen (TASK 3, maximum 40 points). (**B**) Median time to complete and (**C**) median score reading printed cards (TASK 4, maximum 22 points). The boxes here represent the interquartile range. The whiskers comprise all recorded values but outliers. (*p-value ≤ 0.05, **p-value ≤ 0.01, Mann-Whitney-Wilcoxon, + outliers defined as 1.5 the inter-quartile range, n_CONTROL_=4, n_Monocular_=10, n_Binocular_=10)

In TASK 3, regarded previously as ‘pattern recognition and movement detection’, the control group scored 38 ± 0.8 points on average out of a maximum of 40, which represents a 95% success rate. No subject achieved 100% success rate in this task, with 39 points being the highest number of correct observations. As per SBV subjects, the overall scores for the monocular and binocular groups were 26.8 ± 1.7 and 27 ± 3 respectively. Differences between SBV subjects were not statistically significant (p-value = 0.53, *r*=−0.086). However, both SBV performed significantly worse than the control group (p-value = 0.005 monocular, p-value = 0.004 binocular), as shown in Fig. 3A. During this test, participants were allowed to lean as needed to accommodate their visual perception to the screen. Thus, SBV subjects generally leaned towards the screen, likely attempting to align their restricted field of view, limited by the simulated prosthetic implant, with the display area, thus maximizing the number of phosphenes available for the representation of the target. In contrast, control subjects remain in the original position. Note that three SBV-monocular volunteers failed to allocate their field of view to the screen; they were instructed to reposition.

During TASK 4 (two dimensional shape recognition), the total time taken to complete the test and number of points obtained were used as a two different performance metrics. First, on average, the SBV-monocular and binocular groups required the same time (p-value = 0.97, *r* = 0.06) to complete the task, 369 ± 89 s and 370 ± 113 s respectively, as shown in Fig. 3B. Note that among these two groups, the volunteer who completed the test in the shortest time was enrolled in the binocular group (195 s) while the subject who required the longest was enrolled in the monocular group (570 s). As per the control group, these subjects required, overall, less time to complete the test: 65.5 ± 6.4 s. Differences with the SBV-monocular (p-value = 0.006, *r*=−0.74) and the SBV-binocular (p-value = 0.006, *r*=−0.75) groups were statistically significant. Thus, it can be stated that the non phosphenized group completed the test nearly six times faster than those with simulating prosthetic vision. Secondly, regarding the score obtained in this task, statistically significant differences were observed between the SBV-monocular and SBV-binocular groups (p-value = 0.047, *r* = 0.41), as illustrated in Fig. 3B. This can be understood as a better ability to perceive fine details in the binocular group. Note that the average score obtained by SBV-monocular and SBV-binocular subjects were 16.6 ± 2.9 and 14.3 ± 3.7 respectively, out of a maximum of 22 points. Again, the control group performed significantly better than both experimental groups (p-value < 0.01), with an overall score of 21.25 ± 0.25. Regarding the behavior of the volunteers during the test, it has to be noted that subjects in the SBV groups tended to move the card back and forth, and to bring it in close proximity to the headset, seeking to fit the figure to the field of view thus simulated.

TASK 5 aimed at providing a score to determine the ability of the participants to perceive depth in a static position, a typical feature of stereoscopic perception. Here, the overall number of missing objects was used as the performance metric. Note that all control subjects were able to account for all objects. However, SBV groups missed some of them, with overall scores of 0.8 ± 0.4 for the monocular group, and 0.6 ± 0.7 for the binocular group. While differences were not substantially relevant in magnitude, they were statistically significant (p-value = 0.029, *r* = 0.45). Note that difference with the control group were statistically significant with the SBV-binocular group (p-value = 0.011), but not with the monocular group (p-value = 0.428). Counting mistakes tended to arise from contrast rather than from the position of the objects. Thus, the most frequent error was linked to the light-blue eight and the white sphere placed behind it. This can be appreciated in Fig. 1C.

## Discussion

Bionic vision aims at restoring sight to the visually impaired [12–14]. By implanting an electrode array into the proximity of the retina, it is now possible to elicit sensations of light thus allowing the visually impaired to regain functional vision. While facing specific challenges, bionic vision is following the path of the cochlear implant, as the bionic ear has successfully restored audition in more than one million implantees worldwide [28]. Alone this path, a strategy that has demonstrated excellent result in hearing restoration is the use of bilateral implants [34–37]. However, the use of bilateral retinal implants has not been explored to date. Here, using SBV techniques, we have studied whether a bilateral retinal implantation can benefit visual rehabilitation. Our studied focused on two relevant aspects of visual perception: (1) the ability to use sight to move and interact with the environment, and (2) visual acuity or the capacity to recognize the small details required to identify patterns. To the best of the author’s knowledge, this is the first study to compare monocular and binocular SBV under immersive conditions. This novel approach provides new insights into the potential benefit of bilateral retinal implants for the recipients of bionic eyes. Surprisingly, despite the clinically demonstrated relevance of similar strategies in auditory prosthetics, this topic has remained largely unexplored in the field of visual prosthesis.

In this study, subjects in the SBV-binocular group performed significantly better than those in the SBV-monocular group in all 3D-related tasks, including mobility-related (TASK 1 and 2) and static (TASK 5) tests. Note that, given the low resolution employed in our experiments (120 phosphenes per implant), both groups performed substantially worse than control subjects, as expected. Nevertheless, differences among the SBV groups were not that significant when assessing visual acuity. Note for example that both groups provided almost the same number of correct answers in TASK 3, and required the same time to complete TASK 4. In these tasks, the target was presented within the common work distance, and therefore, a large field of view was not of relevant assistance. In fact, bilateral implants, as simulated here, could even deter the perception of fine details, as convergence/divergence is not enabled. Hence, lack of proper visual vergence may lead to binocular rivalry, a phenomenon in which the brain alternates between conflicting images from each eye, thus reducing the quality of vision. Note we could not determine in these experiments whether participants in the SBV group were blinking an eye to perform visual-acuity tasks as a way to stop an inefficient binocular fusion. This would explain the similar results obtained herein among these two group.

The control group was included in this study to serve as a performance ceiling and to provide a contextual reference for interpreting the relative performance of prosthesis users. It has to be acknowledged that direct comparisons between control and SBV subjects may misrepresent fundamentally different sensory and cognitive conditions, and are not meant to suggest equivalence or clinical expectations. The control group provides a non-clinical benchmark to show the gap between artificial and natural vision. Along these lines, future studies may benefit from including low-vision non-simulated controls, or longitudinal intra-subject comparisons.

While SBV is an excellent tool for predicting the performance of sensory prostheses [50–52, 54, 55], this experimental methodology has relevant limitations. First, the duration of this study did not allow to capture the typical learning process in retinal implantees [67], as the experiments were conducted over two days only. Second, the simulated prosthetic vision implemented in this research employed phosphenes that did not represent those described in the literature [52, 68, 69]. The effects of the upper inhibition threshold [1], the crosstalk among electrodes [3], or the stimulation of passing axons among other relevant implant limitations were not captured either. Note there are other effects that can appear months or years after implantation [67–71]. However, the Shannon information [72] delivered by our SBV system, understood as the total number of bits contained in a phosphenized scene, was similar to that available with current technologies.

We have seen in this psychophysical study that SBV-binocular does not pose significant advantages over SBV-monocular in pattern recognition. Although bilateral implants can theoretically increase the amount of information delivered to the brain about the visual scene, improper stereopsis, flicker or binocular rivalry may hamper the integration of this information, particularly in tasks requiring central vision. Consequently, benefits of bilateral implants may be limited to those aspects of life in which perception of depth is a key factor for success. If these results translate to the clinic, future designs of retinal implants should carefully consider the cost-effectiveness of developing bilateral prostheses. In doing so, the use of eye tracking techniques could be considered to modify the field of view each retinal implant, by acting on the objective of the corresponding cameras, thus enabling vergence in bilateral bionic vision. It has to be noted that the use of two implants may substantially increase the volume of other parts of the device, for instance, the camera or the external electronics. Furthermore, the energy requires to drive to implants will also increase, thus reducing the autonomy of the system.

## Conclusion

The study presented herein provides a comparison between monocular and binocular retinal implants using SBV. The development of bilateral retinal implants may help those awaiting treatment for blindness by improving performance in tasks requiring 3D perception. Here, SBV-binocular subjects performed significantly better that SBV-monocular subjects in spatial perception and mobility tasks, but not in pattern recognition tasks. Thus, the simulation study conducted here indicates that, while substantial benefits can be obtained in activities requiring stereoscopic vision—such as immersive orientation and mobility—the inability to direct gaze or focus both implants on a common point of interest, a form of artificial foveation, appears to be the main limiting factor hampering better results in pattern recognition tasks. Should future generation of retinal implants include bilateral electrode arrays, the systems thus designed should consider mechanisms to implement said artificial foveation to provide a broader benefit to the end users. This study not only highlights a critical design consideration but also uncovers encouraging directions for advancing bionic vision technologies toward more effective and natural visual restoration.
